# Effect of Non-starchy Vegetable Consumption on Weight Status in an Overweight and Obese University Population

**DOI:** 10.7759/cureus.91045

**Published:** 2025-08-26

**Authors:** Urwah Ashraf, Qaisar Raza, Minahil Safdar, Muhammad Barkaat Azam, Rakhshanda Batool, Esha Lnu, Kinza Imran, Abbas Khan, Imranullah Shah

**Affiliations:** 1 Department of Food Science and Human Nutrition, University of Veterinary and Animal Sciences, Lahore, PAK; 2 College of Applied Health Sciences, A’Sharqiyah University, Ibra, OMN; 3 Department of Nutrition and Health Promotion, University of Home Economics, Lahore, PAK

**Keywords:** bmi, fat mass, non-starchy vegetables, obesity, university population, weight status

## Abstract

Background

Obesity is a growing public health problem in both developing and developed countries. Negative health-related outcomes such as type II diabetes mellitus (T2DM), cardiovascular disease (CVD), and certain cancers are linked with being overweight and obesity. Fruits and vegetables provide a protective effect against non-communicable diseases, though there is a lack of data regarding the effect of consuming non-starchy vegetables on weight status and related anthropometric parameters.

Aims

The aim of this study was to investigate the effect of the daily recommended intake of non-starchy vegetables on weight status including body mass index (BMI), fat mass (percent body fat (PBF) and visceral fat (VF)), and waist-to-hip ratio (WHR) in an overweight and obese university population.

Methods

This was a pilot randomized controlled trial (RCT), and it was conducted at a public sector university in Lahore, Pakistan. Data were collected from an overweight and obese university population (including students and teaching and non-teaching staff) with a BMI ≥ 23kg/m^2^. A total of 40 participants (20 participants in the control group and 20 participants in the interventional group) were included. The interventional group was provided with 2-3 cups (250 g) of WHO-recommended non-starchy vegetables, while the control group was only provided with dietary recommendations for vegetable intake. Each individual’s weight, BMI, PBF, VF, and WHR were assessed before and after the trial by using Inbody270 (Inbody Japan Inc., Tokyo, Japan), which works on the principle of bioelectrical impedance analysis. Statistical Package for the Social Sciences (SPSS) version 27 (IBM Corp., Armonk, NY, US) was used to examine the data. Paired and independent t-tests were used to compare changes within and between groups, respectively.

Results

A total of 40 participants were included in the study. Among those, 13 (32.5%) were male, and nearly two-thirds (27 (67.5%)) were female. In the control group, there was a slight increase in weight, BMI, and PBF after the consumption of vegetables, but a slight decrease in VF, though the results were not significant. In the intervention group, there was a significant reduction in PBF (t = 2.36, p = 0.029) and VF (t = 4.19, p < 0.001) after intervention, suggesting a significant improvement in these body composition parameters. The intervention group had a greater PBF (45.3 ± 4.40) before the intervention than the control group (39.4 ± 8.41), with a mean difference of 5.83 (t = 2.745, p= 0.009), and greater VF levels (17.15 ± 3.73) than the control group (14.6 ± 4.14), with a mean difference of 2.55 (t = 2.044, p = 0.048).

Conclusion

Our study concludes that there was a subtle improvement in parameters such as body weight, BMI, PBF, VF, and WHR of the intervention group at the end of the trial, whereas the control group’s participants showed an increase in these parameters except VF, which remained constant at the beginning and the end of the trial.

## Introduction

Obesity is a growing public health problem in both developing and developed countries. Since 1990, the prevalence of adult obesity has increased by over twofold globally. According to the World Health Organization (WHO), one in eight people is now living with obesity worldwide; negative health-related outcomes such as type II diabetes mellitus (T2DM), cardiovascular disease (CVD), and certain cancers are linked with being overweight and obesity [1]. Pakistan, the world's ninth most obese country, is dealing with an epidemic that affects people of all ages, particularly children and women, and is expected to quadruple in the years ahead. The prevalence of overweight and obese adults has reached 30% in Pakistan, of which 21% are overweight and 9% are considered obese [2]. Low consumption of vegetables is linked with an increased risk of non-communicable diseases (NCDs) [2]. These diseases are responsible for 58% of all deaths in Pakistan. Young adults are more prone to weight gain and obesity because of lifestyle and dietary choices. The prevalence of obesity has increased in adults in Pakistan due to many factors such as decreased consumption of vegetables and increased intake of fast food. The prevalence of overweight and obese adults in Pakistani universities differs between men and women. Central obesity in men and women in Pakistani adults in universities has reached 31.4% and 46%, respectively; 16% of Pakistani women have a body mass index (BMI) ≥ 25 kg/m^2^, and 30.5% of Pakistani adult men have a BMI ≥ 25 kg/m^2^ [3]. The WHO Expert Consultation panel proposed in 2002 to lower BMI cut-off points to initiate public health action for Asians, classifying BMI = 23-27.5 kg/m^2^ as overweight and ≥27.5 kg/m^2^ as obese [4].

When energy intake exceeds energy expenditure over an extended period of time, obesity develops (determined by the basal metabolic rate, thermogenic impact of meals, and cost of physical activity). Genetic predisposition or background is one factor contributing to obesity. However, lifestyle factors including poor eating habits and physical inactivity might have a greater impact on the startling rise in its incidence [5]. A significant risk factor for the emergence of chronic diet-related disorders, including heart disease, stroke, type 2 diabetes, and several malignancies, is being overweight. One important variable that may be changed to prevent chronic diseases is nutrition and, more especially, the amount of fruits and vegetables consumed [6].

In the past few years, guidelines for fruit and vegetable consumption have evolved [7]. Different studies showed that the consumption of vegetables in Pakistan is 1-2 servings per day, including starchy vegetables, whereas the WHO and Food and Agriculture Organization (FAO) reports recommend adults to consume at least 2-3 cups of vegetables per day (250 g), excluding starchy vegetables [1]. Adequate eating of fruits and vegetables is recommended by both international and national dietary standards as a means of preventing the development of NCDs. It is said that eating a lot of fruit and vegetables can help control weight since they are high in water and fiber and low in energy. Dietary fiber (non-starch polysaccharides) and physical activity were considered to protect against obesity, whereas high intakes of energy-dense but low-micronutrient foods, combined with sedentary lifestyles, were considered as risk factors for obesity [8]. It is hypothesized that the satiating qualities of fruits and vegetables serve as the mechanism via which the consumption of foods that are high in energy and low in nutrients is reduced, hence reducing the total amount of calories consumed [9]. The DASH (Dietary Approaches to Stop Hypertension) and Mediterranean diets give evidence for vegetables' function in controlling weight when combined with a balanced diet. Such diets promote a high consumption of fruits and non-starchy vegetables while limiting other dense in energy, nutritionally deficient meals [10]. Recent research has demonstrated that several natural dietary components can alter body weight and offer a viable method for preventing obesity. According to a study, bioactive components in the diet serve as antioxidants and anti-inflammatory agents by enhancing thermogenesis and energy expenditure while decreasing oxidative stress, hence promoting weight loss. Polyphenol-rich foods such as fruits and vegetables, in particular, can help prevent the physiological increase in weight in the general population gradually [11]. Vegetable consumption may promote better fullness, lessen appetite, and need less energy intake due to their high fiber and water content and low energy density [6].

Research gap

There are no specific studies found on the effect of vegetable intake on the nutritional status of a university population in Pakistan.

Study objective

The study objective is to assess the relationship between non-starchy vegetable consumption and its effects on an overweight and obese university population (including students and teaching and non-teaching staff).

Implications of the study

The implication of the study is to develop targeted nutrition interventions among a university population to reduce the prevalence of overweight and obesity in Pakistan.

## Materials and methods

Design and population

This was a pilot randomized controlled trial (RCT) and was conducted among the university population (including students and teaching and non-teaching staff) in the University of Veterinary and Animal Sciences (UVAS), Lahore, Pakistan.

Sample

A total sample of 40 individuals with a BMI ≥ 23 kg/m^2^ was selected randomly. The study consisted of two groups: one was the control group, and the other was the interventional group. Each of these groups consisted of 20 overweight and obese individuals with a BMI ≥ 23 kg/m^2^ according to South-Asian cut-off points [12]. The interventional group was provided with a variety of non-starchy vegetables based on WHO recommendations for 10 weeks, while the control group was only given guidelines on the WHO daily recommendations for the intake of vegetables.

Inclusion and exclusion criteria

Overweight and obese individuals with a BMI ≥ 23 kg/m^2^ and students and teaching and non-teaching staff of the UVAS are included in this study, whereas normal weight and underweight individuals with a BMI < 23 kg/m^2^ and non-UVASians (students and teaching and non-teaching staff) are excluded.

Randomization

**Figure 1:**
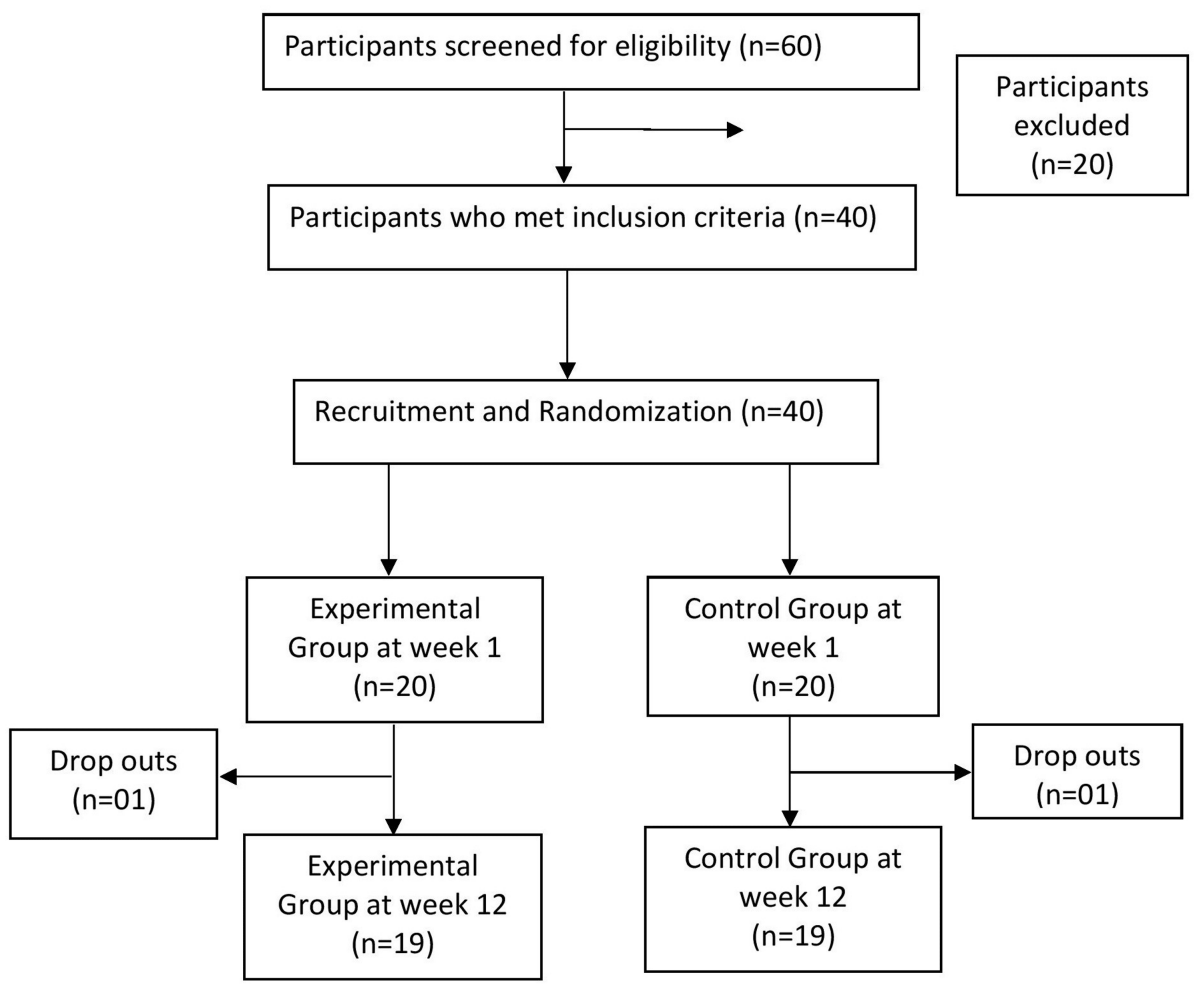
Flow chart of study participants

A total of 40 participants were recruited for the study. Participants were allocated to the treatment and control groups using a randomization technique. Randomization was done by allotting numbers to participants on the basis of the sequence of enrollment. We used a random number generator table to assign a random number to the participant. Then, they were randomly allocated to the treatment group and the control group. Figure 1 shows the number of participants from the start to the end of the trial. At the start of the trial, there were 20 participants in each group, but at the end of the trial, 19 were present in the control group and 19 were present in the experimental group.

Ethical approval

This study (number: 330/IRC/BMR) was approved by the Institutional Review Committee for Biomedical Research of the UVAS, Lahore, Pakistan. This Institutional Review Committee/Biomedical Research (IRC/BMR) is undertaken in compliance with the guidelines set out by the Institutional Review Committee for Biomedical Research. Prior to the commencement of the study, all participants provided their informed written consent to participate. Ethical approval was also taken from www.clinicaltrials.gov under the ID number: NCT06757556.

Study period

The study was conducted for a period of three months (December 2024 to February 2025), and the assessments were done as follows.

Procedure

The pre-assessment, including measurements such as weight, BMI, percent body fat (PBF), visceral fat (VF), and waist-to-hip ratio (WHR), for both the control group and the interventional group was done in the first week. In addition, dietary counselling regarding weight loss pertaining to the WHO dietary recommendations for the intake of non-starchy vegetables was given during the pre-assessment phase. From the second week, recommended raw non-starchy vegetables were provided to the interventional group daily for 10 weeks. The post-assessment of both groups was done in the 12th week.

Vegetables

Our study included non-starchy vegetables such as cucumbers, kale, cauliflower, spinach, cabbage, tomatoes, capsicum, broccoli, and lettuce, which are seen to have profound effects on leptin levels and ultimately reduce the weight and BMI of overweight and obese individuals. Participants of the intervention group were provided with non-starchy vegetables according to the WHO recommendation of daily intake to incorporate into their daily routine.

Study parameters

This study involved measurements such as weight, BMI, WHR, and fat mass (PBF and VF) at baseline and at the completion of the study. The details of the parameters that were measured are described.

Anthropometric measurements

Baseline measurements of anthropometric parameters including weight and BMI, WHR, VF, and PBF were assessed for all participants (a total of 40 participants) using Inbody analysis (Inbody270, Inbody Japan Inc., Tokyo, Japan), which is a body composition analyzer that works on bioelectrical impedance analysis (BIA) to accurately measure different components of the body and a height scale to accurately measure a person’s height. These assessments were obtained at the end of the interventional period as well.

Dietary measurements

Participants of the intervention group were asked to keep a food diary, and that food record was used to obtain the daily non-starchy vegetable intake of individuals and the way they used to incorporate or consume vegetables daily.

Statistical analysis

Data were analyzed with the help of statistical software SPSS version 27 (IBM Corp., Armonk, NY, US). Descriptive statistics were used to present frequencies and percentages of baseline measurements. The Shapiro-Wilk test was used to assess the normality of the data. The Wilcoxon test was applied to data that did not know normality. The paired t-test was applied to compare changes within groups, and the independent t-test to compare differences between the control and intervention groups. Throughout the study's duration, alterations in weight status and fat mass were observed during follow-up visits for both the control group (participants not undergoing dietary interventions) and the treatment group (participants undergoing dietary interventions) over a span of three months. We compared changes in weight status and fat mass using paired sample t-tests at each follow-up visit of both the control group and the treatment group. The statistical significance of the findings was determined by analyzing p-values. In this study, a p-value less than 0.05 was considered significant, indicating that the observed changes in weight status and fat mass were likely due to the dietary intervention rather than random chance.

## Results

A total of 40 participants were included in the study. Among 40 participants, 13 (32.5%) were male, and nearly two-thirds (27 (67.5%)) were female. The study comprised two groups: the control group and the interventional group. Each group consisted of 20 participants. One participant went missing from each control and intervention group due to their failure to attend follow-up and post-assessment appointments. Consequently, the results are based on 38 participants: 19 from the control group and 19 from the interventional group.

Pre- and post-assessment of body composition parameters

Normality of Data

Table 1 demonstrates that the Shapiro-Wilk test was used to check the normality of data. Data were considered normally distributed if the p-value was greater than 0.05 and considered skewed if the p-value was less than 0.05.

**Table 1:** Comparative analysis of normality of data for body composition parameters SD: standard deviation; BMI: body mass index; PBF: percent body fat; VF: visceral fat; WHR: waist-to-hip ratio

Variables	Before intervention			After intervention		
	Mean ± SD	Shapiro-Wilk value	p-value	Mean ± SD	Shapiro-Wilk value	p-value
Control group						
Weight	77.2 ± 14.05	0.921	0.117	78.1 ± 13.48	0.935	0.218
BMI	28.05 ± 3.88	0.920	0.832	28.4 ± 3.95	0.990	0.999
PBF	39.07 ± 8.45	0.973	0.115	39.7 ± 8.84	0.888	0.029
VF	14.47 ± 4.22	0.920	0.114	14.2 ± 3.57	0.930	0.173
WHR	0.96 ± 0.07	0.943	0.300	0.96 ± 0.065	0.878	0.020
Interventional group						
Weight	74.06 ± 11.03	0.938	0.247	71.80 ± 12.3	0.902	0.053
BMI	29.7 ± 4.39	0.967	0.723	29.04 ± 4.66	0.948	0.361
PBF	45.64 ± 4.23	0.926	0.147	44.4 ± 4.73	0.920	0.113
VF	17.52 ± 3.42	0.953	0.444	16.15 ± 3.37	0.918	0.105
WHR	0.96 ± 0.064	0.980	0.939	0.95 ± 0.069	0.971	0.790

Table 1 shows that by applying the Shapiro-Wilk test on the control group (before and after intervention), all the parameters such as weight, BMI, PBF, and VF exhibited normal distribution by showing a p-value > 0.05 except post-assessment of WHR, which showed a p-value < 0.001, demonstrating that the data of WHR (after intervention) were skewed and did not show normal distribution. However, all the body composition parameters of the interventional group (before and after intervention) showed that all the parameters of the data exhibited normal distribution.

As the data of body composition parameters weight, BMI, PBF, and VF of the control group and the intervention group (before and after intervention) showed normal distribution by using the Shapiro-Wilk test, the paired sample t-test was applied on normally distributed data to compare two related samples. In the control group, there was a slight increase in weight, BMI, and PBF but a slight decrease in VF, though the results were not significant. In the intervention group, there was a significant reduction in PBF (t = 2.36, p = 0.029) and VF (t = 4.19, p < 0.001) after intervention, suggesting a significant improvement in these body composition parameters. However, a slight decrease was shown in weight and BMI, but these differences were not statistically significant.

As the data of body composition parameter WHR of the control group after intervention were not normally distributed, the Wilcoxon signed rank test was applied on skewed data to compare two related samples. Table 2 demonstrates that according to the Wilcoxon signed rank test, the z-value (-0.807) showed that measurements of WHR are small and the difference was not far from zero. A p-value of 0.420 was greater than the significance value, which is <0.05, indicating that there was no significant difference between the WHR before and after interventions of the control group.

**Table 2:** Comparison of paired sample t-test for body composition parameters in study groups Data are presented as the mean ± standard deviation (SD). Statistical analysis was conducted using a paired t-test to compare values before and after the intervention within each group. The following parameters are included in the table: mean difference, t-statistic (t), p-value, and 95% confidence interval (CI) A p-value < 0.05 was considered statistically significant BMI: body mass index; PBF: percent body fat; VF: visceral fat; WHR: waist-to-hip ratio

Variables	Mean ± SD		Mean difference	t	p-value	95% CI
	Before Intervention	After Intervention				
Control group						
Weight	77.2 ± 14.05	78.18 ± 13.4	-0.97	-1.84	0.081	-2.09–0.13
BMI	28.05 ± 3.88	28.40 ± 3.95	-0.35	-1.70	0.105	-0.78–0.08
PBF	39.07 ± 8.45	39.7 ± 8.84	-0.63	-1.29	0.212	-1.67–0.39
VF	14.47 ± 4.22	14.26 ± 3.57	0.21	0.39	0.695	-0.89–1.31
Intervention group						
Weight	74.0 ± 11.03	71.8 ± 12.3	2.26	1.91	0.072	-0.22–4.75
BMI	29.7 ± 4.39	29.0 ± 4.66	0.66	1.43	0.169	-0.308–1.63
PBF	45.6 ± 4.23	44.4 ± 4.73	1.24	2.36	0.029	0.13–2.35
VF	17.5 ± 3.42	16.1 ± 3.37	1.36	4.19	<0.001	0.68–2.05
WHR	0.96 ± 0.064	0.95 ± 0.069	0.01	3.00	0.008	0.004–0.02

Table 3 demonstrates that two factors before intervention indicate statistically significant differences between the control and intervention groups. The intervention group had a greater PBF (45.3 ± 4.40) before the intervention than the control group (39.4 ± 8.41), with a mean difference of 5.83 (t = 2.745, p = 0.009) and greater VF levels (17.15 ± 3.73) than the control group (14.6 ± 4.14), with a mean difference of 2.55 (t = 2.044, p = 0.048), whereas only one variable remained significant after intervention. The intervention group showed higher values (44.4 ± 4.73) than the control group (39.7 ± 8.84) after intervention, with a mean difference of 4.68 (t = 2.039, p = 0.049). There were no statistically significant differences between groups in weight, BMI, WHR, or VF (p-values all >0.05) (Table 4).

**Table 3:** Comparative summary of the sample Wilcoxon signed rank test for the study composition parameter Data are expressed as the mean ± standard deviation (SD). The Wilcoxon signed rank test was used to compare pre- and post-intervention values within the group Z^a^ values are reported based on negative ranks ^a^Wilcoxon signed rank test ^b^Based on negative ranks A p-value < 0.05 was considered statistically significant WHR: waist-to-hip ratio

Variables	Mean ± SD		Z^a^	p-value
	Before intervention	After intervention		
Control group				
WHR	0.962 ± 0.07	0.965 ± 0.065	-0.807^b^	0.420

**Table 4:** Comparative analysis of the independent sample t-test for body composition parameters in study groups Comparative analysis using the independent sample t-test for body composition parameters between study groups at pre- and post-assessment. Data are expressed as the mean ± standard deviation (SD). The analysis reports mean difference, t-statistic (t), p-value, and 95% confidence interval (CI) for each variable A p-value < 0.05 was considered statistically significant BMI: body mass index; PBF: percent body fat; VF: visceral fat; WHR: waist-to-hip ratio

Variables	Mean ± SD		Mean difference	t	p-value	95% CI
	Control group	Intervention group				
Pre-assessment of the control group with the intervention group						
Weight	76.5 ± 14.0	73.3 ± 11.16	-3.155	-0.788	0.291	-11.2–4.95
BMI	28.0 ± 3.78	29.5 ± 4.34	1.515	1.175	0.247	-1.09–4.12
PBF	39.4 ± 8.41	45.3 ± 4.40	5.83	2.745	0.009	1.53–10.12
VF	14.6 ± 4.14	17.15 ± 3.73	2.55	2.044	0.048	0.024–5.07
WHR	0.96 ± 0.06	0.95 ± 0.06	-0.003	-0.160	0.847	-0.47–0.04
Post-assessment of the control group with the intervention group						
Weight	78.1 ± 13.4	71.8 ± 12.3	-6.38	-1.52	0.136	-14.8–2.10
BMI	28.4 ± 3.95	29.0 ± 4.66	0.636	0.454	0.653	-2.20–3.48
PBF	39.7 ± 8.84	44.4 ± 4.73	4.68	2.039	0.049	0.02–9.35
VF	14.2 ± 3.57	16.15 ± 3.37	1.894	1.682	0.101	-0.39–4.17
WHR	0.96 ± 0.065	0.95 ± 0.069	-0.014	-0.649	0.520	-0.058–0.030

## Discussion

The aim of the current study was to investigate the effect of consumption of non-starchy vegetables on weight status in an overweight university population in Lahore, Pakistan. This study included 40 overweight individuals. Majority of the participants were women. This current study observed the weight, BMI, PBF, VF, and WHR of the control group and the intervention group (before and after intervention). The current study showed that improvements in weight status were observed in participants who consumed the daily recommended non-starchy vegetables. Participants who received intervention for 12 weeks showed improvements in weight, BMI, PBF, VF, and WHR. However, it is also pertinent to mention that the changes in weight and BMI were predominantly non-significant, while the changes in PBF, VF, and WHR were statistically significant. In our study, regarding WHR, although a within-group reduction was observed in the intervention arm, the lack of statistical significance in the between-group comparison may be attributable to baseline variability and the modest sample size.

In our study, PBF, body weight, and VF showed a statistically significant reduction among the intervention group. Similarly, an RCT was conducted in Brazil, and it showed a 1.4 kg loss in body weight among the intervention group [13]. Our findings showed subtle improvements in body weight in the intervention group (although they were non-significant) after consuming 250 g of vegetables for 12 weeks. Similarly, a study conducted in Japan in 2021 showed that for every 100 g per day increase in overall vegetable consumption, there was an average 25 g drop in body weight. This study showed an inverse relationship between vegetable consumption and weight change [6]. While our study showed a subtle decrease in parameters such as body weight, BMI, PBF, VF, and waist circumference after 12 weeks of intervention, a study conducted to look into relationships between anthropometric indices (weight, BMI, PBF, and waist circumference) and alterations in the intake of fruits and vegetables observed that all anthropometric parameters decreased significantly after 10 weeks [14].

Our research findings showed that there was an inverse relationship between increased intake of non-starchy vegetables and the body weight of participants. While a similar prospective cohort research showed that higher consumption of non-starchy vegetables was associated with weight loss, and higher consumption of starchy vegetables was linked with weight gain in participants [15].

Our research on the effect of consuming non-starchy vegetables on weight status and fat mass revealed that after an intervention of 12 weeks, participants in the intervention group showed a subtle decrease in body weight, and the control group with no intervention showed an increase in body weight. A study conducted with over four-year intervals showed similar results in association with a plant-based diet rich in vegetables and weight status. Over four years, an increase in intake of a plant-based diet that is high in whole grains, fruits, and vegetables was linked to a 0.68 kg reduction in weight gain [16].

Our study showed results of a subtle change in BMI in individuals after dietary intervention, and also concluded that increased vegetable consumption was linked with a reduction in BMI in participants who participated in physical activity and consumed non-starchy vegetables according to WHO guidelines. Similarly, a study carried out in Nigeria evaluating eating habits, physical activity levels, and prevalence of overweight and obesity showed an association between intakes of vegetables and BMI. The study concluded that high vegetable intake was associated with low BMI [17].

Our study was conducted to evaluate the association of consuming non-starchy vegetables and VF. Results showed a significant reduction in VF in participants consuming vegetables as recommended by the WHO. Meanwhile, a similar study was carried out in Japan to look at how eating vegetables high in carotenoid content affected the VF levels of obese Japanese men. The findings showed that consuming more of these vegetables raised plasma carotenoid levels and significantly decreased intra-abdominal VF, indicating a potential benefit in controlling the fat buildup associated with obesity [18].

The study’s findings can be used to guide future researchers, healthcare practitioners, and policymakers to generate evidence and future interventions for health-related benefits and the consumption of conventional and exotic non-starchy vegetables in relation to NCDs. Implementation of dietary intervention programs, counselling, and provision of cost-effective vegetables will help improve the overall health parameters of overweight and obese individuals.

There are a few limitations to this study that need to be addressed in the field of research. These limitations are that biochemical indicators that may offer more detailed information on the metabolic alterations linked to vegetable consumption, such as blood glucose levels, lipid profiles, and inflammatory markers, were not examined in this study. In addition, potential confounding factors such as changes in the dietary habits of individuals and physical activity levels can be potential confounders.

The investigation was conducted during a rather brief three-month timeframe. More clear findings about the long-term effects of eating non-starchy vegetables on weight control could come from longitudinal studies. There can be an increased sample size in future studies to make it more generalizable to the population. In addition, a bigger sample size in future studies might increase statistical power and yield more reliable findings.

## Conclusions

This study concluded that daily recommended consumption of non-starchy vegetables showed improvements in weight and BMI in individuals, though they were statistically non-significant. The participants who received intervention for 12 weeks showed significant improvements in PBF, VF, and WHR. In comparison, the control group participants who did not receive any recommended vegetables showed increased weight, BMI, PBF, and VF at the end of the trial, while the WHR of the control group remained almost constant. Our study had a rather small sample size as it was a pilot study, but future studies should have a larger sample to improve the effect and generalizability of the results.
